# A c.1244G>A (p.Arg415Gln) mutation in SH3BP2 gene causes cherubism in a Turkish family: Report of a family with review of the literature

**DOI:** 10.4317/medoral.19496

**Published:** 2014-03-08

**Authors:** Ahmet E. Sekerci, Burhan Balta, Ying Hu, Ernst J. Reichenberger, Osman A. Etoz, Sinan Nazlim, Ibrahim S. Bayrakdar

**Affiliations:** 1 Assistant Professor, Department of Oral and Maxillofacial Radiology, Faculty of Dentistry Erciyes University, Turkey; 2 Department of Genetics, Faculty of Medicine, Erciyes University, Kayseri, Turkey; 3 University of Connecticut Health Center, Department of Reconstructive Sciences, Center for Regenerative Medicine and Skeletal Development, Farmington, CT, USA; 4 Associate Professor Department of Oral and Maxillofacial Surgery, Faculty of Dentistry Erciyes University, Turkey; 5 Department of Pathology, Faculty of Medicine, Erciyes University, Kayseri, Turkey; 6 ResearchAssistant, Department of Maxillofacial Radiology, Faculty of Dentistry, Ataturk University, Turkey

## Abstract

Objectives: The present study was aimed at advancing the understanding of the pathogenesis of cherubism by presenting a case study based on history, physical examination, typical radiological features, molecular and histopathological laboratory tests and a review of the literature. Study Design: This study began with a 7-year-old boy who was referred due to mandibular overgrowth. A panoramic radiograph revealed multilocular radiolucent lesions of the upper/lower jaws suggestive of cherubism. Overall, a total of four family members were tested for SH3BP2 mutations, namely two siblings and their parents. Both siblings had been clinically diagnosed with cherubism; however, the parents were clinically normal. Peripheral blood was collected from all participants and genomic DNA sequencing was carried out. Results: A missense mutation was found in the two affected siblings and their asymptomatic mother. The mutation was a 1244 G>A transversion which resulted in an amino acid substitution from arginine to glutamine (p.Arg415Gln) in exon 9. Conclusions: The present study emphasized the importance of further clinical and molecular investigation even when only a single case of cherubism is identified within a family. Genotype-phenotype association studies in individuals with cherubism are necessary to provide important insights into the molecular mechanisms associated with this disease.

** Key words:**Cherubism, mandible, maxilla, SH3BP2, gene analysis, CBCT.

## Introduction

Cherubism, which was first described by Jones (1) in 1933, is a rare non-neoplastic and hereditary self-limiting fibro-osseous disease with excessive bone resorption and multilocular lesions in the upper and/or lower jaws during childhood. Affected children are observed to be normal at birth and have normal mental development. Bone expansion becomes noticeable in early childhood and continues progressively until puberty, when the lesions usually resolve at the beginning of adolescence (2). The phenotype ranges from asymptomatic to severe mandibular and maxillary overgrowth with respiratory, speech, vision, and swallowing problems (3). Some observations suggest that by the age of twenty, lesions spontaneously regress, first in the maxilla and then in the mandible (4). Facial appearance may return to almost normal by the fourth or fifth decade of life. However, some patients request surgical recontouring of their residual deformity (5).

Radiologically, the lesions appear as well defined bilateral multilocular radiolucencies, often beginning near the mandibular angle and spreading to the mandibular ramus and body (5). Additional lesions may involve maxillary tuberosities and lesions can spread to all areas, including the maxillary processes (6). The lesions are painless and more or less symmetrical. Bones other than the jaws are not usually affected (7,8).

Several family reports from different countries have clarified that cherubism is a hereditary bone disease (1,9-11) which has no relation to race (7,10-15). Genetic research has mapped cherubism to chromosome 4p16.3 (16). Furthermore, point mutations in the SH3BP2 gene were demonstrated in 12 of 15 families (17). However, other reports have shown that cherubism may occur without familal history suggesting either de novo mutations in SH3BP2 or genetic heterogeneity (14,15). Cherubism is a very rare disorder with only an estimated 300 cases reported in the literature. Because of its rarity, it is difficult to determine a disease frequency for this disorder (18).

The purpose of this study was to determine whether a mutation in the SH3BP2 gene was the molecular basis of cherubism in a Turkish family. This case report describes the history, physical examination, typical radiological features and laboratory (molecular and histopathological) tests and performs a review of the literature.

## Study Design

**Figure 1:**
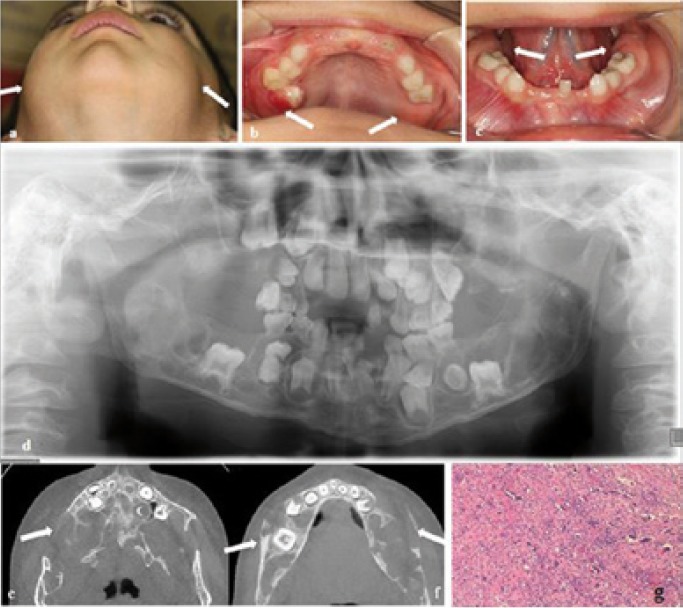
Clinical view of the proband (patient IV1) with cherubism showing prominent swelling on both sides (a) and intraoral view of the patient (b, c). Orthopantomogram showing multiple radiolucencies in mandible and maxilla (d). Axial CBCT images at the level of the maxilla (e) and mandible (f) display grossly expansile cystic lesions involving both bones with cortical scalloping. Typical histopathology of cherubism. A histological section from the cherubism lesion demonstrates the typical finding of multinucleated osteoclast-like giant cells (arrows) near bone and within soft fibrous stroma. Original magnification 200x. (g).

This study began with a 7-year-old (Figure 1: patient 1) boy who was referred due to overgrowth of the mandible. The proband and participating family members (ID numbers: 1,2,3,4) were clinically evaluated by extraoral and intraoral examination, pano-ramic X-rays and cone beam computed tomography (CBCT).

The study protocol was approved by the Institutional Review Boards of the University of Connecticut Health Center and Erciyes University Medical School and informed written consent was obtained from the participating subjects. For the mutation identification, approximately 10 mL peripheral blood was collected from all participants and genomic DNA was extracted using a Gentra Puregene Blood Kit (Qiagen, Santa Clara, CA).

## Results

-Study Cohort

**Figure 2:**
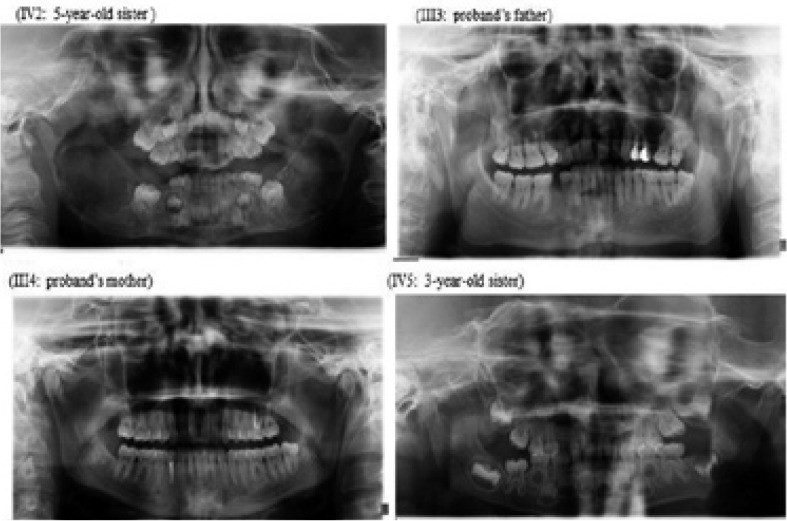
Panoramic radiographs of proband’s family members. Cherubism patient IV2 and clinically and radiographically normal little sister (IV5) and parents III3 and III4.

A total of four family members were tested for SH3BP2 mutations, namely two siblings and their parents. Both patients had been clinically diagnosed with cherubism; however, the parents were clinically normal. Patient 1 (IV1) is a seven-year-old boy who showed mild facial swelling and a delayed permanent first molar in the lower jaw. In patient 2 (IV2) who is a five-year-old girl, an osseous defect and multiple symmetrical cysts in the mandibula and maxilla had been detected. She showed more swollen facial characteristics compared to her brother. The proband also complained of swelling of the right posterior maxillar region. Extraoral examination showed bilateral non-tender enlargement of the lower and upper jaws giving a chubby appearance to both cheeks (Fig. 1). Intraoral examination revealed an inflammatory mucosa around the maxillary permanent first molar tooth in the right maxillary region (Fig. 1) and multiple swellings in the posterior maxillary and mandibular regions (Fig. 1) which were sessile, hard, non-tender and covered by pink mucosa. Blue sclerae were also observed. Panoramic radiograph (Fig. 1), and the cone beam computed tomography (CBCT) images (Fig. 1) showed bilateral, multilocular, expansile-radiolucencies with cortical thinning and destruction within the ramus, angulus and corpus of both sides of the mandible. Similar lesions were also noted in the maxilla. A bone biopsy from the mandible was consistent with giant cell granuloma (Fig. 1). The members (parents and two sisters) of the proband’s family were invited to our dental department. His sister (patient IV2), who is a five-year-old girl, presented with a painless symmetrical progressive swelling of the cheeks. Intraoral evaluation of the posterior maxillary and mandibular molar region revealed bone expansion, which was firm and hard to palpation with intact overlying mucosa. A panoramic radiograph (Fig. 2) revealed bilateral multilocular, expansile radiolucencies of the mandible, particularly in the symphysis of the mandible. The lesions spared the mandibular condyles but involved the maxillary tuberosities. In the panoramic radiograph the developing tooth germs for the permanent second premolars were not visible. Clinical and radiographic evaluation of other members of the family (father III3, mother III4, sister IV5) was normal (Fig. 2).

-Mutation Identification in SH3BP2

Exon 9 of SH3BP2 was amplified from genomic DNA by Polymerase Chain Reaction (PCR) using primers designed with Primer 3 (http://primer3.sourceforge.net). Amplifications with primers 5’-CTTGCCGTCCTCACACAGAG-3’ and 5’-TTAGGAACTGTGGAGTCCTG-3’ were performed in 20 µL reactions containing 20 ng of genomic DNA, 4 mL 5x PCR buffer, 0.4 mL 10 mM dNTPs, 1.6 mL 25 mM Mgcl2, 8 µmol of each primer, and 1U Taq DNA polymerase (Promega, Madison, WI). Unincorporated primers and dNTPs were removed by treating the PCR product with Exosap-IT according to the manufacturer’s instructions (Affymetrix, Santa Clara, CA). PCR products were sequenced on an ABI PRISM 3730 automated sequencer (Genewiz, South Plainfield, NJ) and resulting sequences were compared to Genebank reference sequence NM_001145856.1.

**Figure 3:**
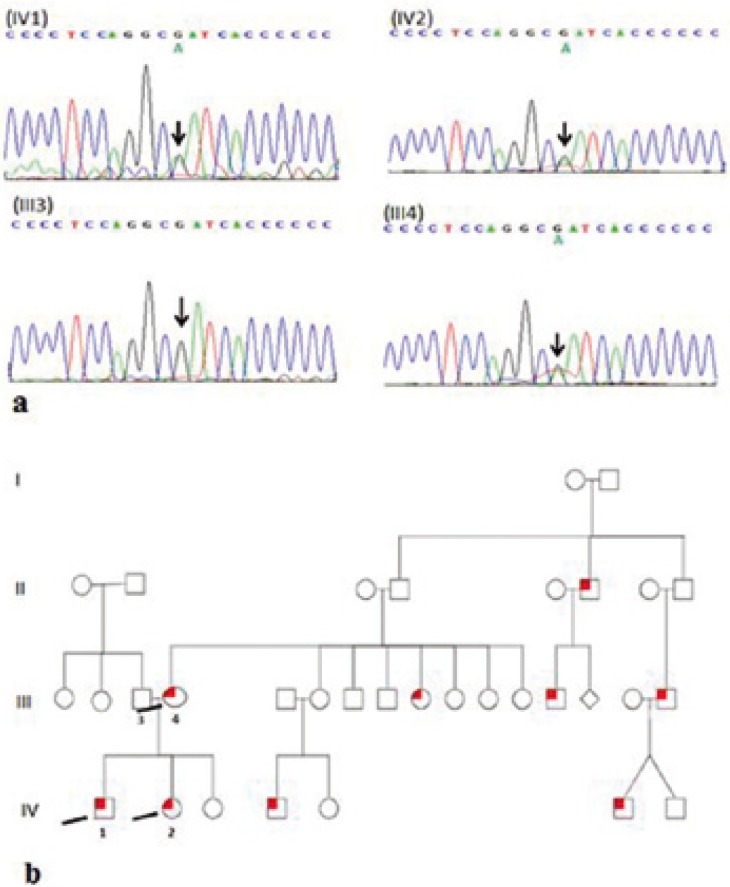
Pedigree of CBM family included in the study. Pedigree showing autosomal-dominant inheritance of CBM. Individuals who were tested by Sanger sequencing are indicated by arrows (a). SH3BP2 mutations in GENOMIC DNA. The electropherograms show 1244 G>A transversion in SH3BP2 of 2 patients (IV1: proband and IV2: middle sister), the unaffected father (III3) and the clinically normal but genotypically affected mother (III4) (b).

We found a missense mutation in the proband IV1, the affected sibling IV2 and their asymptomatic mother III4 by Sanger se-quencing, which has been previously used for cherubism. The mutation was a 1244 G>A transversion which resulted in an amino acid substitution from arginine to glutamine (p.Arg415Gln) in exon 9. The asymptomatic mother came from a big family in which at least six people were affected with cherubism. The father had a wild type genotype and no family history of cherubism. It appears that the mother is an obligate carrier and transmitted the mutation to the two affected offspring (Fig. 3).

## Discussion

The hallmark of cherubism is the development of symmetrical multilocular radiolucent expansile, non-neoplastic, self-limiting fibro-osseous lesions in the jawbones (12). Bilateral painless swelling tends to begin within a range of 14 months to 12 years of age, usually within the first few years of life. In the present study, bilateral swellings were first noticed when children were about three years old. It should be noted that, during the late teens, the disease may undergo spontaneous, gradual, partial or complete involution and that older patients with a mild form of cherubism may have bone lesions that have been remodeled with normal mandibular bone and therefore signs of cherubism may no longer be detected by radiographs (7). In the present study, the diag-nosis of cherubism was based on patient age, family history, clinical examination, radiographic findings, and molecular analysis.

The clinical presentation ranges from minor lesions without facial deformity to large, deforming, destructive lesions with massive involvement of both jaws (6,17). In more severe forms of cherubism, the fibro-osseous tissue extends into the inferior and/or lateral orbital walls. The skin over the cheeks is stretched and pulls down the lower eyelids. Physical displacement of the globe and retraction of the eyelid results in exposure of a rim of the sclera beneath the iris. In the present study, both of the patients affected by cherubism have blue sclera. The disease may also invade the retrobulbar spaces of the orbits and cause displacement of the optic nerves and proptosis (13). Significant ocular disturbances, such as lid retraction, proptosis, superior globe displacement and diplopia may occur (5). There have been reported cases of cherubism with massive enlargement of the jaws and backward displacement of the tongue or obliteration of the nasal airway resulting in airway obstruction and obstructive sleep apnea, mouth breathing, snoring, chronic nasal infection speech, in addition to mastication and swallowing problems (3). Some patients with severe cherubism report episodic pain (3,13).

The first radiographic signs of cherubism are usually found in the region of the mandibular angle and spread to the ascending rami and body of the mandible. The radiographic and computed tomography signs of cherubism are as follows: expansile bone remodeling, cortex thinning, scalloping and disruption, multilocular radiolucencies with a coarse trabecular pattern and soft-tissue density within, dental anomalies, absence of periosteal reaction, and sparing of the condyles (19,20). Magnetic resonance imaging (MRI) examination is useful in elucidating the accurate extent of the cherubic lesions, particularly their relationship to the orbits and the optic nerves (4). These radiolucent lesions are asymptomatic but may affect the development or eruption of the permanent molars. The more progressive form of cherubism manifests with multiple symmetrical lesions in the mandible or involves the mandible and maxilla with singular or multiple lesions (21). By 30 years of age, lesions are frequently not detectable. In a follow-up study of 18 patients with cherubism, von Wowern found progressive new bone formation in the lesions of patients over 20 years of age (22). By 41 years of age, the bone structure in the affected areas was completely normal. Diagnosis in adults with a mild form of cherubism, not appreciated in childhood, can be difficult as lytic bone cysts fill in with bone and may not be radiographically detectable. However, in rare instances actively expanding lesions in suspected cherubism may be diagnosed in adults (23). Carvalho et al. reported that the cherubism lesions in 7 of their 8 patients stabilized by the age of 12 years and regressed thereafter (5). One more severely affected patient showed features of cherubism at the age of 20. Radiographic examination at follow-up visits revealed filling of the radiolucent lesions with bone as early as 2 years after stabilization and in most patients when they were in their twenties. In the present study, the children presented with typical bilateral facial swelling and radiographic evidence of cherubism.

As cherubism is generally a self-limiting condition and regresses with age, treatment depends on the clinical course of the disease and is indicated only in the cases of esthetic or functional problems. The treatment of cherubism should be based on the known natural course of the disease and the clinical behavior of the individual case. Most of the cases require no treatment. Most dental surgeons prefer to wait until the end of puberty before performing a surgery (24). In the present study, no treatment option was suggested, the parents were informed about cherubism and the children scheduled for a follow-up appointment. During the growth phase of the lesions, annual clinical and radiographic examination with a panoramic or other appropriate radiograph is recommended. Follow-up every 2 to 5 years is advisable after the disease becomes quiescent. The impact of cherubism lesions on the development and eruption of the primary and permanent dentition varies depending on the time of onset and severity of the expansile lesions. Widening of the alveolar ridges is common. The arrangement of primary teeth can be disturbed (11). Disruption of the secondary dentition can include absent teeth (mostly molars), rudimentary development of molars, abnormally shaped teeth, partially resorbed roots or delayed and ectopically erupting teeth (11). Tooth extraction may be needed, especially if the teeth are “free-floating” in cherubism lesions (10) or if they become ectopically impacted (22). In more severe instances, children may require prostheses that need to be adjusted as the child grows or the swelling within the oral cavity changes. A dental prosthesis may improve the ability to chew and increase the self-esteem of the child. Orthodontic treatment is usually required after growth is completed and when cherubism is regressing (18). Surgical intervention is often necessary for severe functional disturbances (24). Expansion of fibrous lesions in severe cases may regress well after adolescence (15). In other instances actively expanding lesions in suspected cherubism may still be diagnosed in adults. Patients with aggressive, rapidly progressing cherubism should be evaluated by a craniofacial team consisting of a surgeon (oral and maxillofacial surgeon, plastic surgeon, otolaryngologist), geneticist/genetic counselor, ophthalmologist, dentist/orthodontist and child psychologist/ social worker and nurse (18). Papadaki *et al.* (18) reported that mild forms of cherubism without facial dysmorphology, dental or ocular involvement may not require treatment as cherubism is expected to regress spontaneously after puberty. Management in these cases consists of longitudinal observation. Von Wowern reported 18 patients with cherubism who underwent biopsy with or without autotransplantation of ectopically erupted teeth. Surgical treatment did not provoke progression of the lesions in any of these cases (22), which is consistent with other reports (19). Raposo-Amaral *et al.* (20) reported on extensive resection in 8 children aged 6 to 15 years old with severe cherubism. Surgical resection during the proliferating phase of the disorder was performed in 2 stages to prevent excessive blood loss. The maxilla and orbits were contour-resected first and the mandible 6 months later through intraoral and extraoral incisions. The patients were followed-up for 2 to 18 years and no recurrence was found in any of them. Both patients and authors were satisfied with the outcome and the authors suggest that thorough removal of affected tissue appears to arrest the proliferation of any remaining tumor tissue (18). In Etoz et al’s case report, systemic calcitonin therapy was advocated, and favorable results were achieved (19).

Radiotherapy is contraindicated because of fear of retardation of jaw growth, radio osteonecrosis and chances of malignant degeneration (25).

The authors believe that further phenotype-genotype association studies in individuals with cherubism will help in establishing the basis for phenotypic variabi-lity and provide important clues to its pathogenesis. The present study emphasizes the significance of additional clinical and molecular examination even when only a single case of cherubism is identified within a family.
